# Effect of Paraffin and Silica Matrix Phase Change Materials on Properties of Portland Cement Mortars

**DOI:** 10.3390/ma14040921

**Published:** 2021-02-15

**Authors:** Vicente Zetola, Luis J. Claros-Marfil, Alfonso García Santos, Fco. Javier Neila González

**Affiliations:** 1 Departamento de Gestión de la Construcción, Facultad Ciencias de Ingeniería y Construcción, Universidad Católica del Norte, Avda. Angamos 0610, 1270709 Antofagasta, Chile; 2 Departamento de Construcción y Tecnología Arquitectónicas, E.T.S. Arquitectura, Universidad Politécnica de Madrid, Av. Juan de Herrera 4, 28040 Madrid, Spain; luisj.claros@gmail.com (L.J.C.-M.); alfonso.garciasantos@upm.es (A.G.S.); fjavier.neila@upm.es (F.J.N.G.)

**Keywords:** Phase Change Materials (PCM), silica-paraffin microparticles, Portland cement mortars

## Abstract

In the search for methods to incorporate Phase Change Materials (PCM) into Portland cement mortar mixtures, PCM based on paraffins adhered to a silica-based matrix appear as a suitable option. However, paraffin particles have been observed to escape from the silica matrix when water is added. There are only limited data on how the use of such PCM affects the behaviour of mortars. To evaluate the effect of this PCM addition, Portland mortar mixtures were elaborated with 5%, 10% and 15% of PCM content, and using CEM 42.5 I R and CEM I 52.5 R cement types. Physical properties such as density, open porosity, air content and water absorption were analysed for fresh and dry samples. The results obtained show that the PCM-added mixtures require greater water and cement amounts than the standard mortar mixtures to achieve similar compressive strengths. Compared to non-PCM mixtures the PCM-added mortars present a density lowering of 37% for fresh mixtures and near 45% for dry state forms. A maximum compressive strength of 15.9 MPa was reached for 15% PCM mixtures, while values beyond 40 MPa were achieved for 5% PCM mixtures. Thus, the proposed study contributes to broad the available knowledge of PCM cement mortar mixtures behaviour and their mix design.

## 1. Introduction

The energy storage in walls, ceilings and floors can be improved by adding phase change materials (PCM) to construction materials. These PCM are able to increase thermal inertia and indoor thermal stability, to make more efficient use of renewable energy, to allow passive cooling and to enhance the overall thermal performance of buildings [1,2,3]. There is a vast range of available PCM that are classified as organic, inorganic and eutectic materials, depending on its composition and characteristics [4,5,6]. They can be added to construction materials by different methods [7] as direct application, immersion, microcapsules [8,9] and macrocapsules [10,11].

PCM addition to mortar and concrete mixtures has been widely studied. They can be applied through immersion, impregnation in porous aggregates and direct mixing [12]. Some examples are the study of PCM impregnation in light sands [13], the works about PCM impregnation in concrete blocks [14], the use of burnt clay aggregate to improve thermal properties of a concrete panel [15], the research about the effect of light sand impregnated with PCM over the thermal behaviour of concrete during setting and freeze conditions [16], the fabrication of cement-based composite phase change materials (CCPCMs) based on the water solubility of polyethylene glycol (PEG) and the hydraulicity of cement [17] and the development of polyurethane acrylate coated salt hydrate/diatomite form-stable PCM with enhanced thermal stability [18].

On the other hand, research related to the experimental and numerical investigation of thermal properties of cement-based materials modified with PCM for building construction use [19] has been performed, and a review about the potential of microencapsulated PCM for energy savings in buildings has been presented more recently [20]. So far, the applications to Portland cement mortars of PCM based on paraffin nucleus and polymeric shells, have evidenced changes in the mixtures, such as a decrease in resistance, an increase in the amount of water and a decrease in thermal conductivity and an increase in heat capacity [21,22,23,24].

A study about the addition of PCM particles directly to mortars was performed by Cunha et al. [25], verifying good performance in physical and mechanical properties of mortars mixtures. This also happens when PCM are combined with other materials as diatomite [26,27], graphite [28], or silica [21,29]. The effect of microencapsulated phase change materials (MPCM) on the mechanical properties and microstructure of Geopolymer concrete (GPC) and Portland cement concretes (PCC) has also been explored [30]. The results shown as the compressive strength of both GPC and PCC decreased when the quantity of MPCM increased. More recently, the use of forest biomass ashes (FBA) in cement-based mortars was examined [31]. The results have shown that mechanical strengths are slightly reduced, and the ductility is enhanced. Moreover, it has been stated that the incorporation of FBA waste does not affect the scope of the use of these mortars.

There are two kinds of PCM that may be added to Portland cement mortars: protected PCM, which are covered by a stable shell, and non-protected PCM, which are not covered by this type of shell [29]. In this work have been used protected PCM made up from microparticles of dry dust, formed by a silica-based matrix with poly-nucleus of paraffin. They present a severe drawback: silica and paraffin get separated when they contact water, which may lead to a microparticles leakage during the mixing process. Some authors [27] have proposed alternatives to this issue employing three different kinds of nano-silica to modify the PCM. The non-protected PCM present as their main drawback that they can be filtered during the mixing process, or when they are applied in porous materials. At the same time, the lack of a shell in this kind of PCM makes they present the advantage of owning a higher storing heat capacity than inorganic PCM [32]. The direct incorporation of non-encapsulated PCM also allows a cost reduction, due to the absence of a PCM encapsulation process which avoids the need complex incorporation techniques, representing an innovative and promising way to contribute for the energy efficiency of buildings significantly [33,34].

This paper focuses on the mechanical and physical behaviour of protected PCM based on dry dust microparticles, which are conformed by a silica-based matrix with poly-nucleus of paraffin in Portland cement mortars. These mortars were selected due to their capacity to store paraffin particles inside and their compatibility with the silica matrix. Mortars prepared with this kind of PCM own the advantage of improving their heat storage capacity, having a lower retraction, and avoiding problems related to thermal conductivity, which make them appropriated for thermal applications. Contrarily, their main drawback is the possible leak of PCM when using a direct method to be incorporated into the mixture, which has been suggested as the reason for the scarcity of research studies [8].

With the aim of analysing the effect of PCM addition to Portland cement mortars, different mixtures with various PCM contents, water to cement ratios (w/c) and two types of cement were elaborated, and properties such as apparent density, water absorption, open porosity, compressive strength, air content and behaviour analysis after exposure to 35 °C were studied. The gained results have a direct application in the mixing design process since air content, need of water, types of cement, water/cement ratios and compressive strengths are studied.

Additionally, in contrast with other studies, cooling techniques were used to avoid paraffin leaks through the mixing procedure.

## 2. Materials and Methods

### 2.1. PCM Characterization

**Figure 1:**
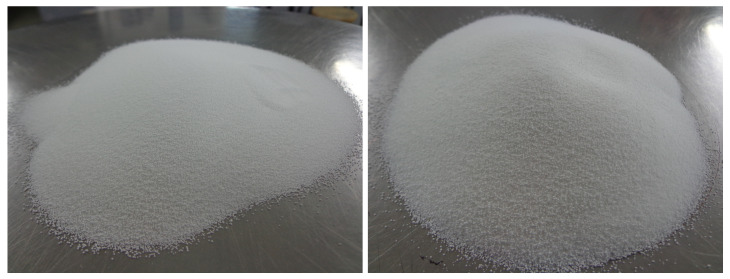
Microencapsulated paraffin based PCM: DS5001 X (**left**) and PX27 (**right**).

The PCM selected were PX27 [35] and Micronal DS5001 X [36] (Figure 1). The former was used mostly in the preparation of mortars, while the latter was employed only to compare the behaviour of mortars probes when heated. A comparative between their physical properties is presented in Table 1.

According to its technical datasheet, PX27 is made up of dry dust microparticles, formed by a silica-based matrix with the poly nucleus of RT27 paraffin particles which have a size of 0.25 mm. The silica included in the dry dust microparticles gets dispersed when it contacts with water during the mixing process. For PX27 the phase change temperature occurs between 24 and 28 °C, its heat-storing capacity has a value of 117 kJ/kg (Table 2), and its relative density is so high as 1100 kg/m^3^.

**Figure 2:**
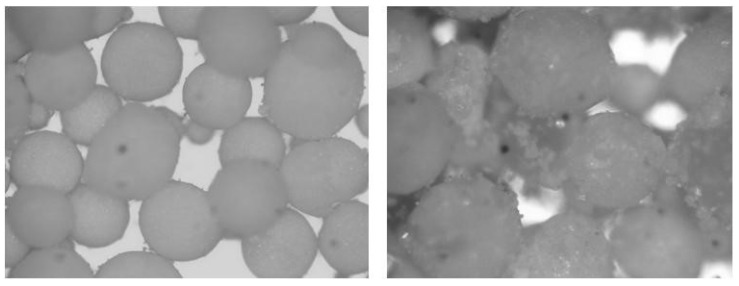
Microscopical view of paraffin based PCM: DS5001 X (**left**) and PX27 (**right**).

Micronal DS5001 X, named as DS5001 X in the tests, is made of dry dust microparticles conformed by mixing microencapsulated paraffin with highly reticulate poly (methyl methacrylate) (PMMA). The individual microparticles with a size of 5 μm (Figure 2) are reticulated in particles with a size between 0.1 to 0.3 mm, which are dispersed along the mixing process. The phase change temperature for DS5001 X is 26 °C, and it owns a latent heat-storing capacity of 110 kJ/kg [36].

### 2.2. Cements Properties

The Portland cements used were CEM I 42.5 R and CEM I 52.5 R types, in accordance to UNE-EN 197-1:2011 norm [37]. Their properties, according to the manufacturer, are summarised in Table 3.

### 2.3. Sand Properties

**Figure 3:**
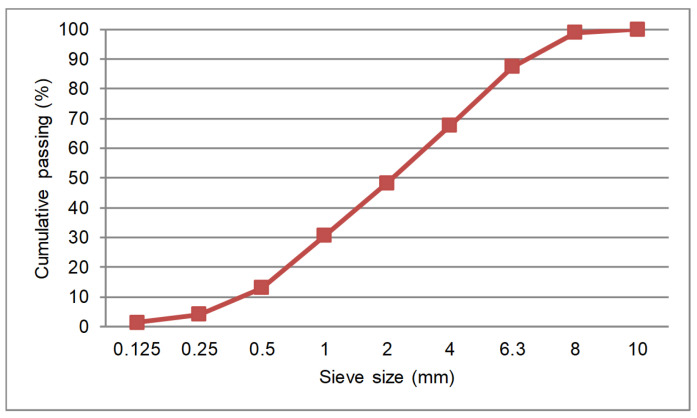
Combined aggregated granulometry of sand.

River sand with 2600 kg/m^3^ dry surface particle density and a 0.47% water absorption coefficient was used. The granulometry curve is depicted in Figure 3.

### 2.4. Superplasticiser Properties

A water-based modified polycarboxilate superplasticiser [38] was used in the samples preparation for improving the mortar workability. Its properties are shown in Table 4.

### 2.5. Tests

Different tests were performed to verify the fresh and hardened behaviour of cement mortars and the PCM dry microparticles. The most relevant aspects related to the thermal behaviour of the PCM were verified and, more precisely, the thermal behaviour along the mixing process and the PCM behaviour until 1000 °C were tested. The first studied aspect was the effect of the silica-based matrix addition to the mixtures for values below and above the phase change temperature. With this aim, the PCM were mixed with water and subsequently heated beyond the PCM melting temperature.

A number of tests were carried out to verify the PCM effect over the fresh mortar behaviour consistency and its apparent density. To get the apparent density of PCM-fresh mortar mixtures the procedure was as follows: a 0.25 L glass container was filled with three mortar layers. Then, these layers were compacted by 15 strikes using a 12 mm squared wood bar and leveraged with a glass plate. Later, the compaction essays were performed on a vibrating table, according to UNE-EN 1015-3:2000 norm [39]. The mixtures preparation for tests was made according to point 6.2 from the UNE-EN 196-1:2005 standard [40], excepting that all the materials were first introduced into the mixing machine and later mixed by hand for 15 s.

Additionally, to verify the PCM influence over some of the hardened mortar mixtures behaviour, the probes were submitted to different tests such as apparent density, water absorption, open porosity, compressive strength, air content and exposure to 35 °C. The test methodology to get the apparent density of the hardened specimens is based on a partial adaptation of the UNE-EN 1015-10:2000 norm [41].

Water absorption and open porosity for low-pressure water test were measured with 1 cm of water above them, using partially the methodology indicated in UNE-EN 1015-18:2003 norm [42]. The formulas used for calculating water absorption (Equation (1)) and open porosity (Equation (2)) were proposed by Mayor Lobo and Hernández Olivares [43], while the real volume of fresh mortar (Equation (3)) and the air content percentage (Equation (4)) were estimated using the methodology adopted in the NCh 1564 of 2009—Annex C Chilean norm [44].

(1) $$W_{a}\left(\%\right)=\frac{P_{S}-P_{d}}{P_{d}}\cdot 100$$

(2) $$O_{p}\left(\%\right)=\frac{P_{S}-P_{d}}{P_{S}-P_{SS}}\cdot 100$$

(3) $$V_{r}=V_{c}+V_{a}+V_{W}+V_{PCM}+V_{d}$$

(4) $$A_{c}\left(\%\right)=\frac{V_{a}-V_{r}}{V_{a}}\cdot 100$$

For the compressive strength testing of mortars with microencapsulated paraffin PCM incorporated the following methodology was used: (1) the kneading process was done as previously explained; (2) the mixture was then poured into parallelepiped moulds (16 cm (span) × 4 cm (width) × 4 cm (depth)), and it was compacted again according to UNE-EN 1015-11:2000 norm [45]; (3) after extracting each part of the mould, it was wrapped by a plastic film until the test time; (4) finally, the compressive strength was gained according to norm UNE EN 196-1:2005 [40]. The compressive strength tests were performed in two different compression test machines depending on the required loads: a Microtest test machine with 200 kN maximum compressive load, and a Mecánica Científica S.A universal compression test machine with 20 kN maximum load. The flexural tension essays were performed in a Microtest test machine equipped with a 10 kN EX-F transducer. A summary of all the used norms to evaluate the mortar mixtures properties is presented in Table 5.

To verify the PCM material-mortar behaviour at temperatures higher than phase change temperature, a complementary set of probes with a 35 cm (span) × 35 cm (width) × 4 cm (depth) size were elaborated and exposed to 35 °C for ten hours.

### 2.6. Mixtures Design and Test Plan

The design of mixtures was defined to achive a plastic consistency of around 160 mm, measured in a shaking table. The superplasticiser admixture (SP) was supplied at the maximum manufacturer dose recommendation, that is 1.5% cement weight, excluding mortars without PCM in which 0.5% cement weight was used. The cement content determination was done according to water quantity, and the w/c ratio used corresponds to the mortar dosages test plan shown in Table 6. The quantity of water considered in the w/c ratio does include the liquid part aggregated by the superplasticiser admixture. The PCM partially replaces the sand, and its percentage is related to the total mixture weight.

The composition of all the elaborated samples is detailed in Table 6. The first column of the table identifies the mixture with an unique ID. The first term of this ID refers to the kind of cement used: CEM I 42.5 R (I42.5) and CEM I 52.5 R (I52.5), the second one refers to the w/c ratio (0.50, 0.70 and 1.0), the third term refers to PCM weight content (0%, 5%, 10%, 14% and 15%), and the last one refers to the PCM used. There are some missing mixtures in the table like I42.5-0.50-15-PX27 and I52.5-0.50-15-PX27 since in these particular cases the physical volume of the raw components makes impossible to incorporate the PCM in practice. The same happens for I52.5-0.70-15-PX27, and due to this, the mixture I52.5-0.70-14-PX27, with 14% PCM, was made instead.

It is worth to mention that the nominal values for the w/c ratio are fixed, and the real values gained are very near (or equal) to the nominal values. The hands-on laboratory experience shows that when a PCM is added to a mortar mixture, the workability will be primarily affected due to the inherent nature of these materials (in this case paraffin). Therefore, in order to reach a minimum workability, the required quantity of water varies, so does the quantity of cement, and consequently the same happens for the quantity of sand. If the quantities of cement and the w/c remain unmodified, the PCM addition will lead to a non-workable mortar mixture. On the other hand, if the quantity of sand is not changed, the resulting mixtures will have different volumes. Additionally, the PCM percentage is then referred to the mixture weight and, as a result, it also varies depending on the mixture density. This means, from a practical point of view, that to elaborate a specific volume of a mortar mixture containing PCM it will be not possible to maintain fixed the quantities of the raw materials, conversely to w/c ratio which indeed remains constant.

### 2.7. PCM Leakage Test

To analyse the occurrence of leakages in the hardened PCM mortar mixtures, two probes with a mild content of PCM (Table 7) were exposed to 40 °C, which exceeds the PCM melting temperature, for twenty-four hours.

## 3. Results

### 3.1. PCM Influence on the Mixture Homogeneity

**Figure 4:**
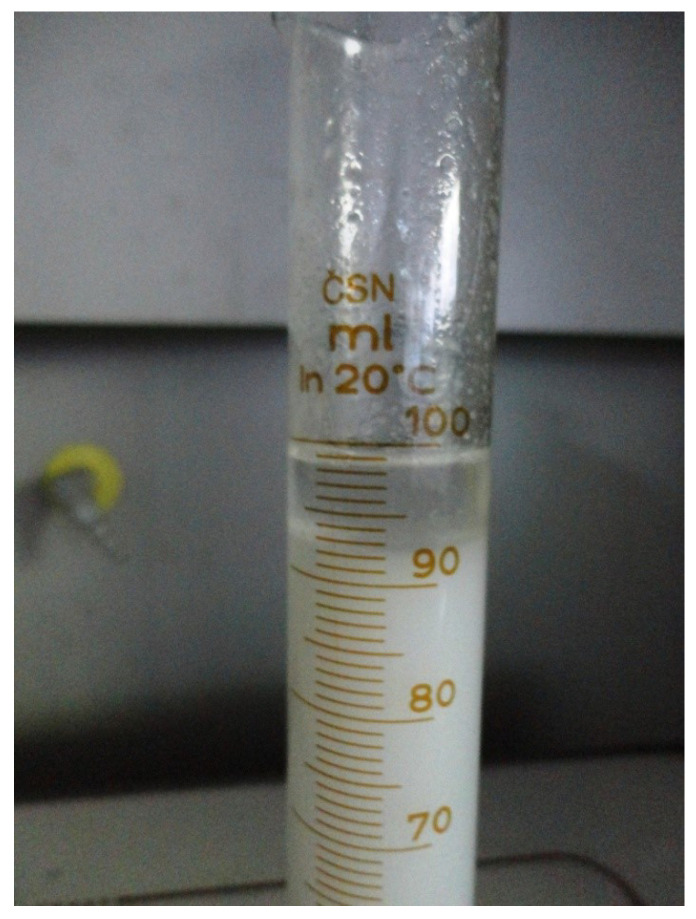
PX27 mixed with warm water.

**Figure 5:**
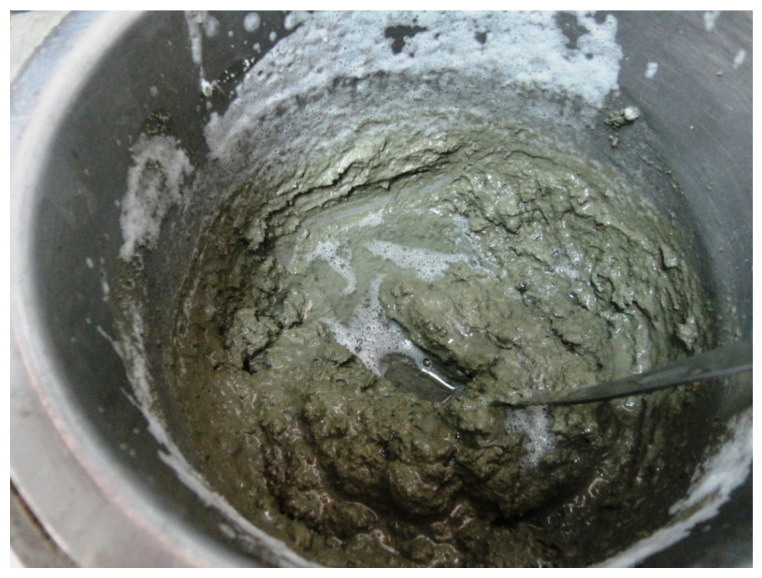
Liquid paraffin on the surface of a PX27 mortar mixture.

With the aim of verifying the behaviour of dry dust particles of RT27 after contacting water at a temperature below phase change temperature, a PCM sample was tested. When the added water is heated beyond the phase change temperature, it is possible to see how liquid paraffin flows to the surface (Figure 4). This suggests that the silica-based matrix and the paraffin micronodules are disaggregated when getting in contact with water warmer than the paraffin fusion point (which is 26 °C for the selected paraffin). As a consequence, the behaviour of the paraffin micronodules depends on the temperature at which the mixing process is carried out. Based on the mixing process observations, three different states are possible from the workability point of view: (1) when the mixing process is done at temperatures below the PCM phase change temperature, no paraffin is observed on the mixture surface during the mixing process; (2) if this mixing process is done at temperatures near the PCM melting point, where a part of the paraffin is changing its state, no paraffin is observed on the mixing surface, but an improvement on the workability is noticed, which suggests that paraffin may act as a plasticiser; (3) finally, at a temperature higher than the paraffin melting point, the mixing process is fully developed, and liquid paraffin appeared on the mixture surface (Figure 5). In order to avoid the appearance of such paraffin, the applied solution was to cool the materials according to the ACI 305 Committee Guide recommendations [46]. By using this procedure, the mixtures were performed without any issue despite the ambient temperature was above the PCM melting point. Therefore, when incorporating PCM in the mortars mixing process, the operation temperature is a key variable to be controlled.

### 3.2. PCM Effect over the Mortar Dosage

According to the dosages for mortars mix design shown in Table 6, the PCM addition to the mixture directly affects the need for the required water. That is, the more PCM is added, the more water is needed, especially when cement CEM I 52.5 R instead of cement CEM I 42.5 R is used. This greater need for water affects all other components of the mixture. Furthermore, for a given w/c ratio, the need for cement increases according to the quantity needed for a non-PCM mortar. This rise for cement need becomes more significant the more PCM is added to the mixture. That is, low resistance mixtures offer better PCM adding possibilities for obtaining a mixture with suitable workability properties.

In PCM mortars mixtures, the PCM replaces the sand. Consequently, the sand content diminishes as PCM content increases. This causes that, at the same time, both the mortar resistance and the hydraulic retention increase. Furthermore, the diminution of sand makes that the mixtures enhance their cohesion forces, which leads to workability problems. It is difficult to set the optimal quantity of sand for these kinds of mixtures, but according to our experience an interval between 800–1200 kg/m^3^ is a proper choice. It must be taken also into account that if sand content is reduced, the hydraulic retention will be increased.

**Figure 6:**
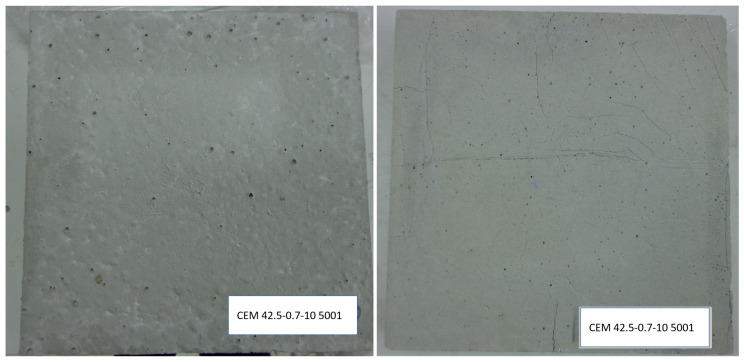
Micronal DS5001 X samples: original probe (**left**) and heated probe (**right**).

**Figure 7:**
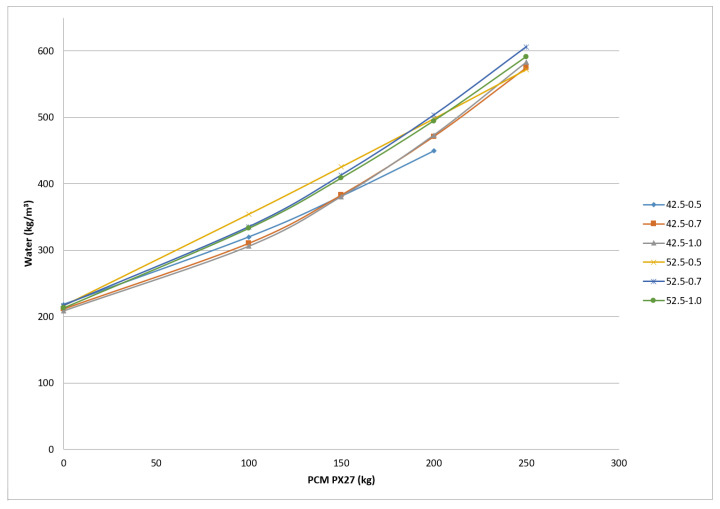
Water content for mixtures with different PCM PX 27 content, cements and w/c ratio.

When the elaborated 35 cm (span) × 35 cm (width) and 4 cm (depth) test probes were exposed to heating and cooling processes between 35–45 °C and later exposed continuously to ambient temperature, some cracks appear on the edges (Figure 6). A summary of the cracks and fissures appeared in the Micronal DS5001 X and PX27 probes is included in Table 8. It has been considered that a crack appears when the damage fully crosses the probe, while a fissure happens when the damage appears only on the probe surface. In relation to the PCM used, for the same w/c relation the cracks and fissures that occur in the probes after subjecting them to heating of around 40 °C, are smaller for PX27 (without cracks), than for Micronal DS5001 X, which present three cracks with a total length of 11 cm. This may indicate that drying shrinkage is lower in PX27 mortars than in Micronal DS5001 X ones. On the other hand, the increase of the PCM content in the mixtures leads to an increase of the water needed for their preparation (Figure 7). This increase is greater for PX27 than for DS5001 X [15]. The difference between the behaviour of the mixes of these two materials are relatively low for mixtures with a 100 kg PCM/m^3^ mixture ratio, but it becomes more relevant for a 200 kg PCM/m^3^ mixture ratio. Such behaviour could be originated by the free silica contained in the PCM.

### 3.3. PCM Effect over the Fresh State Mixture Characteristics

The laboratory work revealed that when water was added to a PX27 mortar mixture, it is split into two parts: silica matrix and poly nucleus of paraffin. The most evident effect of such effect is the reduction of the workability properties of the mortar (measured by a flow table test), which involves the need for more water. An additional consequence, which has been already described, is the change of phase of the paraffin during the mortar mixing process, which depends on the mixing process temperature and the phase change temperature. So, it was observed that at the initial time the mixtures have low workability, but later it is improved. This may be due to a part of the paraffin changes its state from solid to liquid, and this liquid part enhances the mixture workability. In general, the mortar mixtures where PX27 was used presented a suitable workability in the fresh state but offered a low consistency in the shaking table.

Micronal DS5001 X mortar mixtures showed a lowering in its workability when compared to a standard Portland cement mortar. Additionally, due to the paraffin in this PCM is encapsulated, leaks were avoided during the mortar elaboration process, even for temperatures beyond the phase change [21].

**Figure 8:**
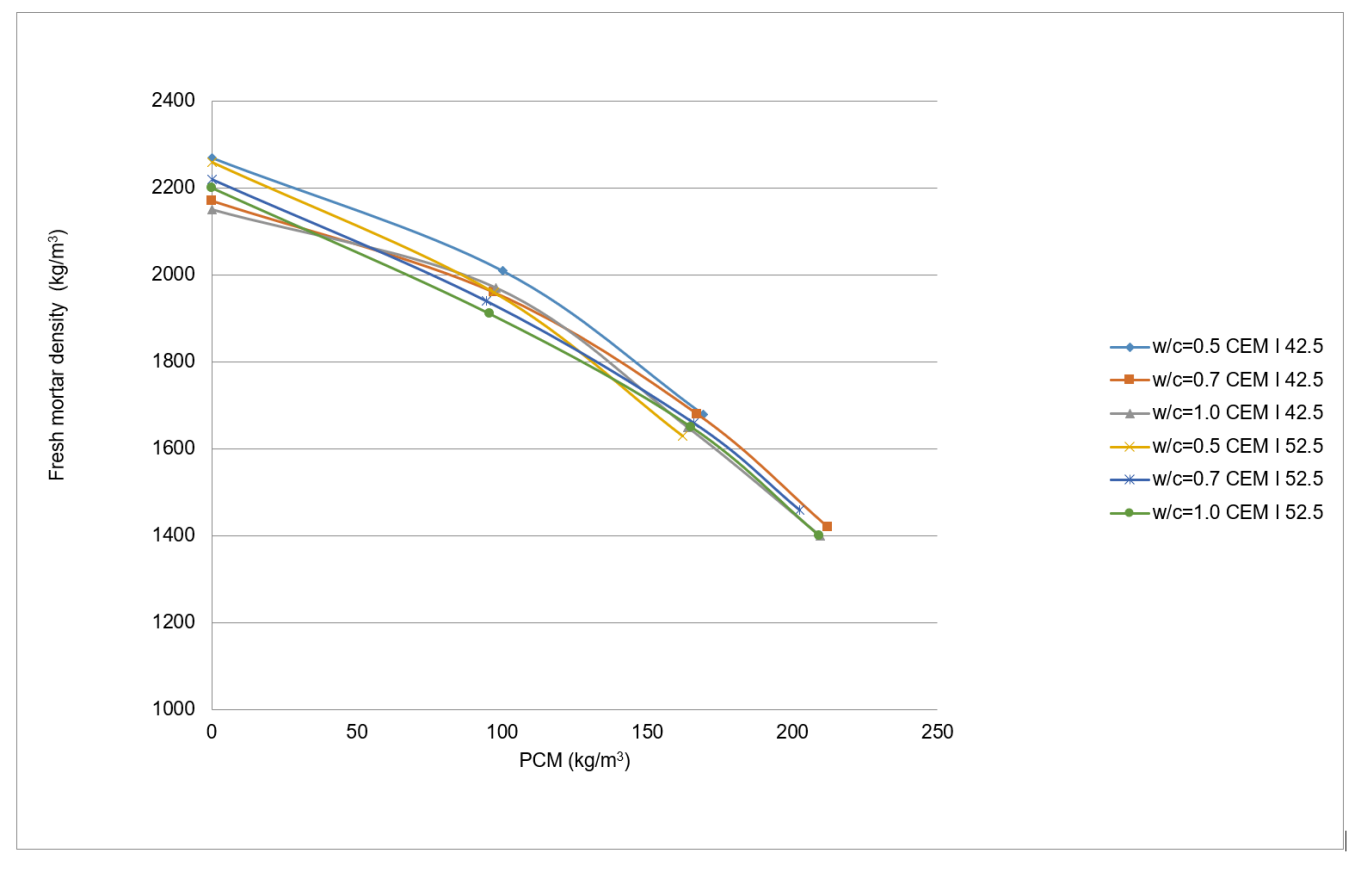
Fresh mortars density variation.

The density values of the fresh mortars for the PX27 mixtures are shown in Figure 8. Each point corresponds to two measurements, and 12 kg/m^3^ maximum standard deviation was gained for 100 kg PCM, w/c = 0.5 and CEM I 52.5 R. It was observed that the density differences are more significant when the PCM content diminishes reaching values around 1400 kg/m^3^ for 210 kg PCM. If the same quantity of Micronal DS5001 X is used, then the density values vary between 1600–1800 kg/m^3^ depending on the w/c ratio [21]. The reason for such behaviour is that water requirements increase with the PCM content (Figure 7). This additional water occupies a greater volume in the mixture and thereby reduce the volume of sand, which cause mixtures with greater density values.

The water content of mortars with microencapsulated PCM may be less than 120 L/ m^3^ mixture [21]. However, the reduction of the water quantity could limit the maximum PCM content which can be added to the mortar. On the other hand, the increase of the water content and the use of the superplasticiser admixture, required to maintain a good workability of the PCM mortars [7], lead to other collateral effects as lowering global density of the mixtures. This is a consequence of the replacement of sand by other materials with a lower density.

**Figure 9:**
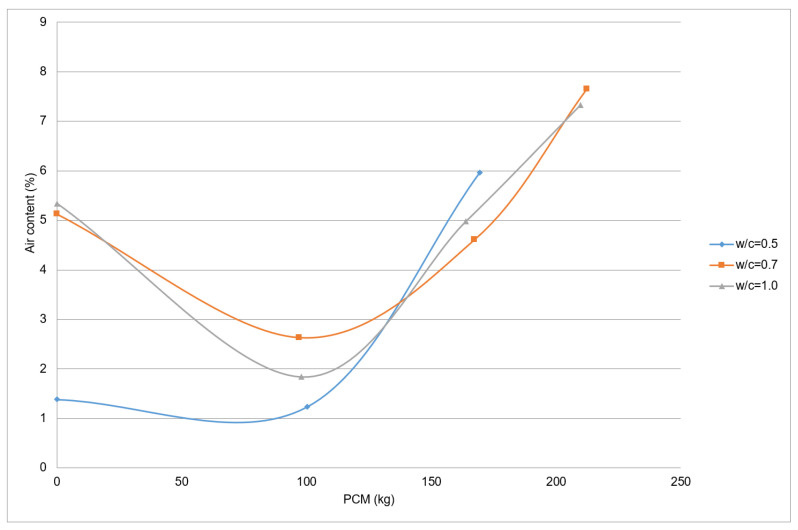
Air content in PX27 and CEM I 42.5 mixtures.

**Figure 10:**
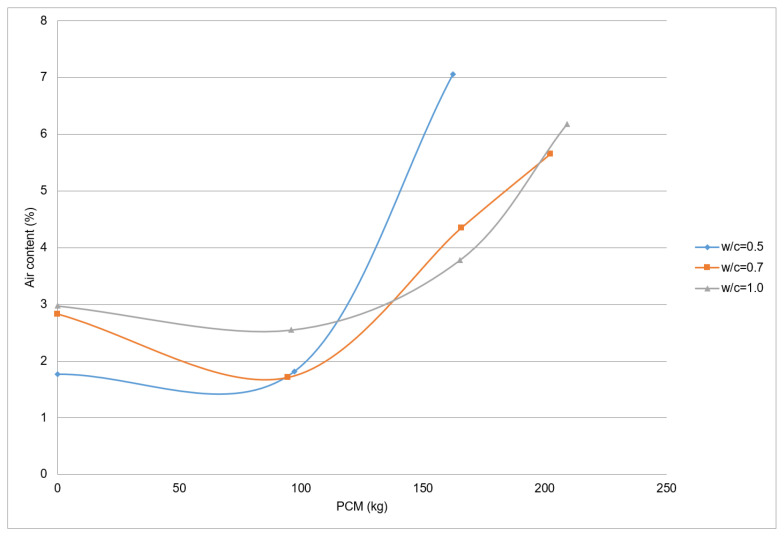
Air content in PX27 and CEM I 52.5 R mixtures.

The relation between the air content of the CEM I 42.5 R fresh mortar, the PX27 and the different w/c ratios are depicted in Figure 9 where each point represent one measurement. It may be observed that when there is no PCM content in the mortars, they present greater or similar air content than mortars with 100 kg of PCM for the same w/c. This behaviour does not happens in CEM 52.5 R mortars (Figure 10), so it would be necessary to develop additional tests to analyse this. Moreover, the mixtures of mortars with PCM show that the more PCM is added, the more percentage of air content appears. Thus, the samples with the greater quantities of PCM added (210 kg) presented the maximum air contents, which are around 7% for w/c = 1.0 and near 8% for w/c = 0.7.

In the case of CEM I 52.5 R and PX27 fresh mortar, the relation between air content and PCM content for mixtures with different w/c ratio is shown in Figure 10, where each point refers only to one measurement. It can be observed as these mixtures show a behaviour similar to the CEM I 42.5 R samples. However, in this case, the mixtures with a w/c relation equal or greater than 0.7 show an air content of around 6% when 210 kg of PCM are used. Furthermore, it can be seen as, in general, 2–3% air content increase in the fresh mortars occurs when more than 150 kg PCM/m^3^ mixture are used.

### 3.4. PCM Effect over the Dry Hardened Mortar Mixtures Characteristics

**Figure 11:**
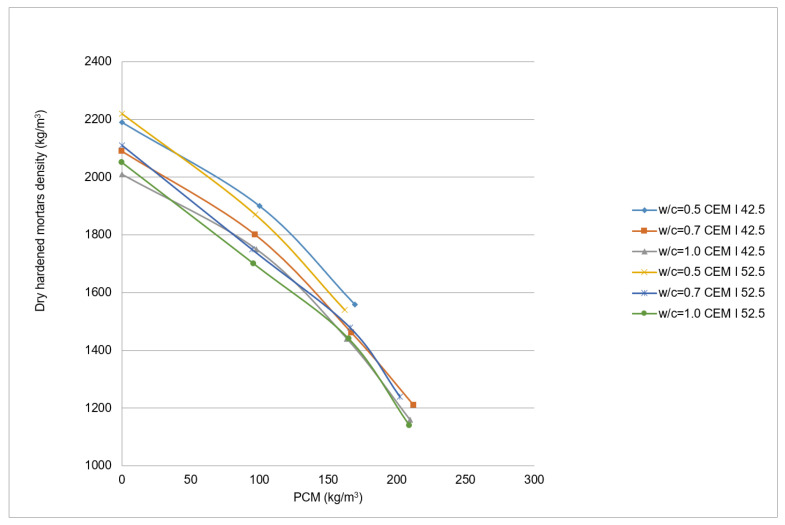
Dry hardened PX27 mortars density variation.

Concerning the effect of the PCM over the dry hardened mortar mixtures, characteristics such as density, water absorption, open porosity and mechanical properties were analysed. The relation between the density of the hardened dry mortars and the PCM content is shown in Figure 11. Each point represent the mean value of three measurements, with 13 kg/m^3^ maximum standard deviation gained for the 5% PCM, w/c = 0.5 and CEM I 52 R point. A similar behaviour than the observed for the fresh mortar case is observed here. Nevertheless, the differences between the density values are greater in this case and, compared to the fresh mortars, the curve is displaced in a considerable proportion when the w/c relations grow. The minimum density values, of around 1150 kg/m^3^, are obtained for mixtures with 210 kg PCM. This means a reduction of near 250 kg/m^3^ compared to the fresh mixes, and it is due to the loss of water along the drying process.

**Figure 12:**
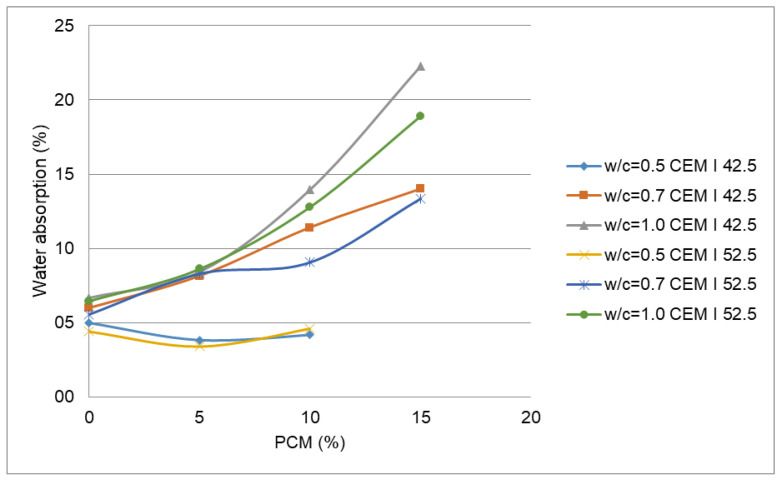
Water absorption in PX 27 mortar mixtures.

**Figure 13:**
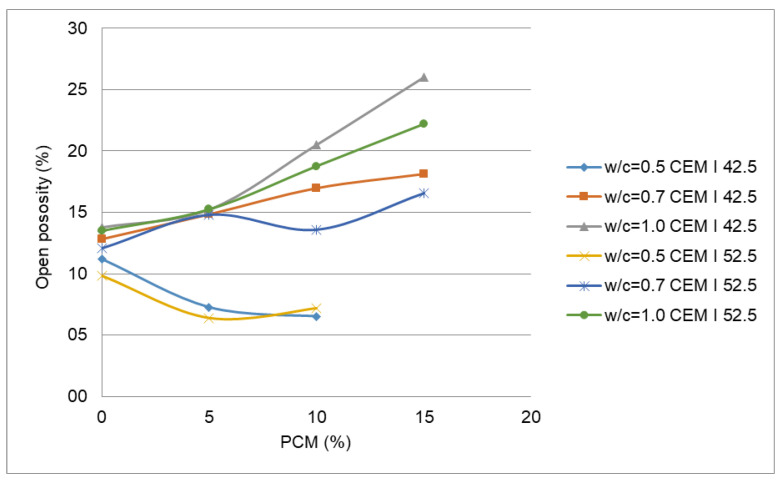
Open porosity in PX27 mortar mixtures.

The effect of water absorption on PX27 mortars mixtures is presented in Figure 12. It must be noted that mixtures with w/c =0.5. and 15% PCM content were discarded due to their poor workability. Each point corresponds to the mean value of three measurements, with 0.5% maximum standard deviation obtained for the point corresponding to 5% PCM, w/c = 0.7 and CEM I 52.5 R. Here it is observed how water absorption increases according to PCM content. For a w/c = 1, the water absorption reaches values between 18% for CEM I 52.5 R and 22% for CEM I 42.5 R. According to the figure, the water absorption decreases when the w/c ratio decreases for both types of cement. In this manner, for 10% PCM and w/c = 0.50 absorption goes close to 5% for both types of cement, but for w/c = 1.0 it grows up to 12.8% for CEM I 52.5 R and 14% for CEM I 42.5 R. The mixtures with w/c = 0.5 presented a good behaviour respect of water absorption, the same as mixtures with no PCM. However, it is observed as the more the PCM content increases the more the water absorption grows. The same effect happens for open porosity (Figure 13). Therefore, the increase of the water content and w/c ratio increase water absorption and open porosity values. Some authors suggest that the diminution of the density values may be caused by an increase in the PCM mortar porosity [8].

In relation to open porosity values for PX27 mortars, they are achieved for the highest w/c ratios (Figure 13). Each point represents the mean of three measurements, and 1.1% maximum standard deviation is gained for 5% PCM, w/c = 0.7 and CEM I 52.5 R. When w/c = 0.5 are used, open porosity values are around 7%, which represents a reduction compared to mortars without PCM that owns a value of 11%. For w/c = 0.7 the open porosity values are around 14%, this is slightly higher than the values obtained for the mortar without PCM. Finally, when w/c = 1 the open porosity values increase almost linearly with the PCM quantity.

The PCM quantities added to the mortars for the different kinds of cement and w/c relations are summarised in Table 9.

**Figure 14:**
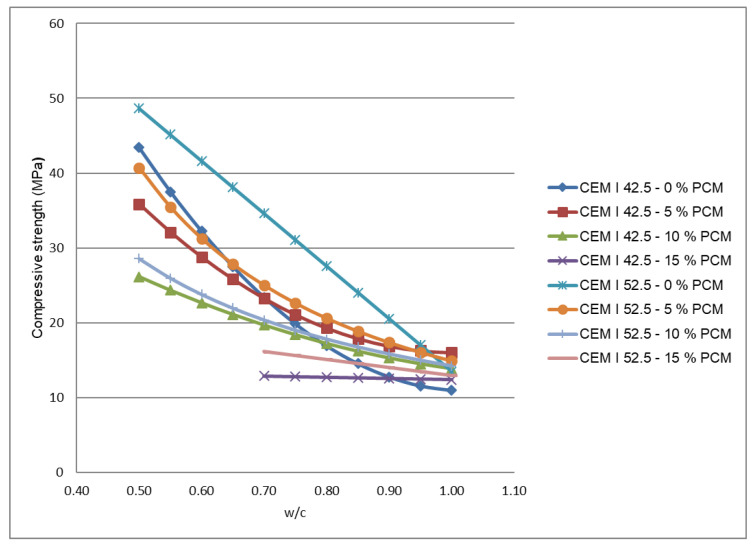
Compressive strengths in PX27 mortar mixtures.

The compressive strength values change according to PCM percentages for the studied mortars is shown in Figure 14, where different curves for each type of cement and PCM content are presented. Each point corresponds to the mean of six measurements, and a maximum 2 MPa standard deviation is obtained for 5% de PCM, w/c = 0.5 and CEM I 42.5 R. It is shown as for a given w/c ratio and PCM content the compressive strength is greater when CEM I 52.5 R cement is used. Moreover, regardless of the used cement, the compressive strength achieved values are greater as w/c decreases. It also can be observed how the compressive strength differences between the PCM mortars and non-PCM mortars decrease as the w/c ratio grows. This difference is reduced to zero when CEM I 52.5 R and w/c = 1 are used, and may reach even negative values if CEM I 42.5 R and w/c = 0.8 are employed. It must also be considered that, due to the increase of water use, the PCM mortars mixtures will need a greater quantity of cement to achieve the required w/c ratio.

The compressive strength obtained for the w/c = 0.5, 5% PCM and CEM 52.5 I R mixture was so high as 41 MPa, while 16 MPa was achieved when w/c = 1 was used instead. This contrasts with the 8–25 MPa compressive strengths reported by other authors for these mixtures [8]. In the case of 10% PCM, w/c = 0.5 and and CEM 52.5 I R, a 28 MPa compressive strength was obtained. This result is similar to the one proposed by Zetola et al. for microencapsulated mortars [21], but lower than the 32 MPa value reported by other authors [8].

**Figure 15:**
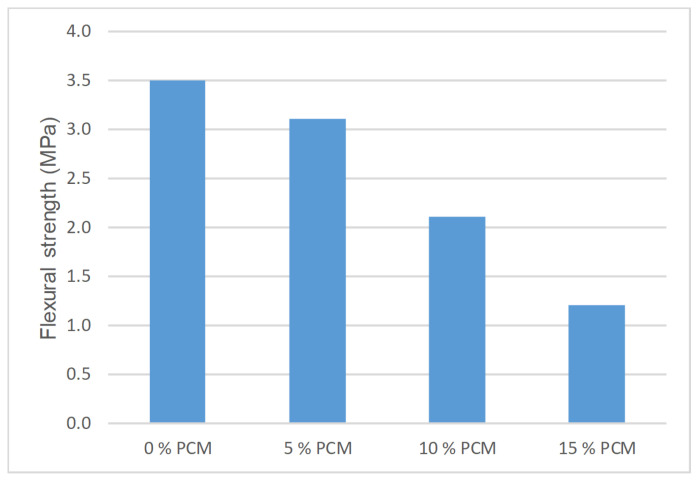
Flexural strength for PX27, CEM I 52.5 R and w/c = 1 mortar mixtures.

The flexural strength results for PX27 are reproduced in Figure 15. Each point corresponds to the mean value of three measurements, while 0.12 MPa maximum standard deviation was gained for w/c = 1, 10% PCM content and CEM I 52.5 R. As it is observed, using until 5% of PCM does not cause a significant lowering of strength. In contrast, the addition of 10% and 15% of PCM causes the reduction of flexural strength by half.

### 3.5. Effect of Temperature on Hardened Mortars

**Figure 16:**
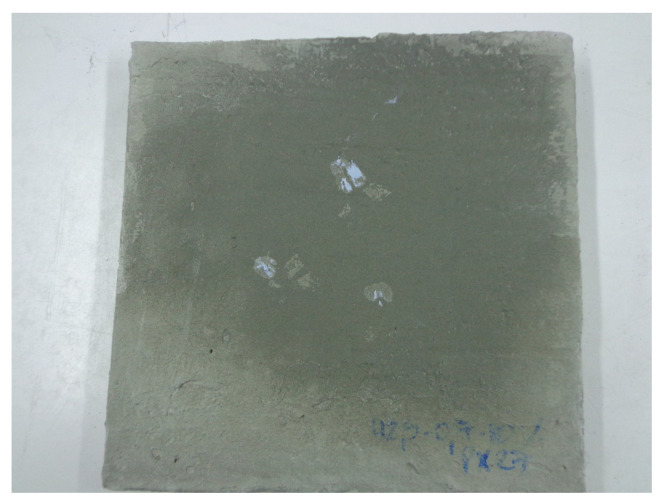
Heated PX27 hardened test probe.

To verify the effect of temperature in hardened PX27 mortars, a 35 cm (span) × 35 cm (width) and 4 cm (depth) test probe was heated to 40 °C for twenty-four hours and then cooled to ambient temperature. This process caused the probe test surface turned darker, but even in this case, no PCM leaks were observed on the sample surface (Figure 16). Since this test was performed only for a 10% PCM and w/c = 0.7 ratio mortar mixture, it would be desirable to perform complementary tests, especially for mortars with w/c = 0.5 which presented lower open porosity and water absorption.

## 4. Discussion

### 4.1. PCM Behaviour in the Mortars

The effect of PX27 addition to the mortar mixtures is influenced by the separation of the paraffin micronodules and the silica-based matrix. This may be deduced from the workability results gained from the mixtures prepared in the laboratory at temperatures above and below the melting point of the PCM. The low density of the paraffin nodules makes possible that, if the fusion temperature is exceeded, the paraffin nodules may flow as liquid paraffin to the mortar surface when they are released from the silica matrix.

In order to avoid this effect, the mixtures must be prepared under a controlled temperature ambient, and always working below the PCM change state temperature by using the appropriate cooling procedures. These techniques consist mainly in cooling the mortar components, according to the ACI 305 Committee Guide recommendations [46]. In this case, the raw materials were cooled in a refrigerator up to 8 °C, which enabled the completion of the mixing process below the PCM change state temperature. Once the mortar is in hardened state, paraffin spots may appear on the probe surface. However, the run-off of liquid paraffin on the surface of the mortar has not been observed.

At the same time, the silica and the free paraffin nodules cause some changes on the workability and the physical characteristics of the mixtures in fresh state. So, a higher quantity of water is required as the PCM content increases in the mixture. This need for water becomes more significant when CEM I 52.5 R is used, and generally makes necessary the use of superplasticisers. As a result, the added water does affect to all the other components of the mixtures and to the physical and mechanical properties of mortars.

It was also noted that mortars which include this silica-based PCM are less prone to cracking than PCM mortars based on methyl methacrylate (MMA) shell microcapsules and paraffin nucleus.

### 4.2. Effect of the PCM on the Physical Characteristics of the Mortars

#### 4.2.1. Density of the Mortars Mixtures

**Figure 17:**
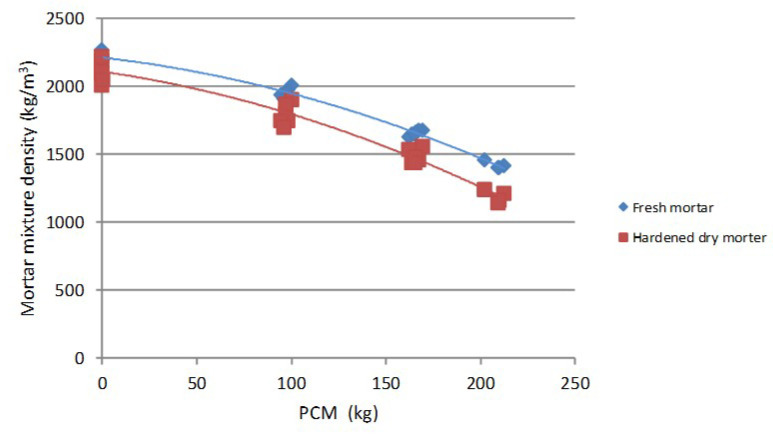
Density comparison of PX27 fresh and hardened mortars mixtures.

It has been observed as the density of the fresh mortars diminishes as the PCM content increases (Figure 17). Each point correspond to the mean of six measurements until 10% PCM mixtures, and to the mean of four measurements for 15% PCM mixtures. The fresh density maximum standard deviation is so high as 43 kg/m^3^, and it is reduced to 25 kg/m^3^ for the 15% PCM content mixture. The dry density maximum standard deviation increases to 74 kg/m^3^, but this value is reduced to 40 kg/m^3^ for the 15% PCM content mixture. The mentioned tendency is also followed by dried mortars, despite the density decrement is partially due to the drying process in these samples. The decrease in the density values between fresh and dry mortars vary around 160 kg/m^3^ mean value (9%). By using Figure 17, it is possible to estimate the density of the mixtures according to the quantity of PCM added.

#### 4.2.2. Air Content, Water Absorption and Open Porosity

The addition of a maximum of 100 kg PCM to the mortar mixtures reduces the air content. This may happen due to the silica particles dispersion, which acts as fines and improve the mixture compactness. In contrast, when greater quantities of PCM are used, the air content will tend to increase.

**Figure 18:**
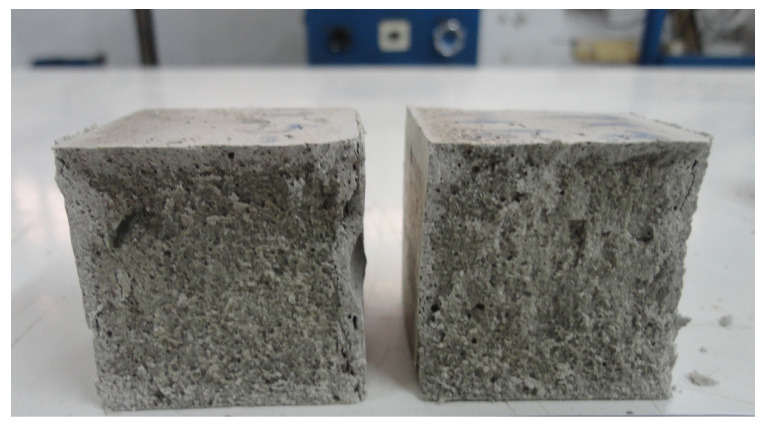
Section of 15% PX27 mortar probe.

In hardened mortars with w/c = 0.5 and 10% PCM content, the water absorption and the open porosity get reduced respect to mortars without PCM. In general, for greater w/c ratios, the more quantity of PCM is used, the more absorption and the open porosity both raise. The increase rate of these variables becomes more evident when high contents of PCM and CEM I 42.5 R cement are used (Figure 18). The rise of water absorption (Figure 12) and open porosity (Figure 13) are influenced by the elevated water quantities that require mortars with a high content of PX27.

### 4.3. Effect of PCM Addition on the Mechanical Characteristics of the Mortar

The mechanical tests performed showed that compressive strengths diminish when a PCM is added to Portland cement mortars. These compressive strengths decrease as the w/c ratio increases. The reason for this could be the reduction of water and cement quantities in the mixture, and the increase of sand quantity. This behaviour reverts in the case of mixtures where a CEM I 42.5 R cement, w/c ratio greater than 0.8 and 10% maximum percentage of PCM are used. The same happens when a CEM I 52.5 R cement, w/c ratio greater than 1.0, and a maximum content of 15% PCM are employed. In any case, it must be considered that, depending on the used cement, the water requirements increase may vary from 100 to 140 L for 100 kg PCM, and even greater quantities for higher PCM contents. Consequently, the PCM mortars mixtures need greater quantities of cement to achieve compressive strengths analogous to non-PCM mortars.

In relation to the maximum compressive strengths, the gained results for the different quantities of added PCM are summarised in Table 10. It is observed as using 5% PCM in mortars with CEM I 42.5 R and CEM I 52.5 R lead to 36.2 MPa and 41.3 MPa maximum compressive strengths respectively. When this PCM percentage rises to 15% the compressive strengths fall to 12.4 and 13.0 MPa for CEM I 42.5 R and CEM I 52.5 R, respectively. Therefore, for a fixed w/c ratio, the greater compressive strength is gained when CEM I 52.5 R cement is used.

Otherwise, as it can be observed in Table 10, compressive strengths of around 80% of a non-PCM mortar strength value can be achieved by mixtures with 5% PCM. Moreover, using CEM I 42.5 cement and w/c = 1 the compressive strengths of 15% PCM mortars are even greater than the obtained for non-PCM mortars.

If mortars with high compressive strengths and a high content of PCM are required, mixtures with high quantities of water and cement, and a low content or total absence of sand will be needed. In particular, the mortars with high compressive strength and a high content of PCM presented high contents of silica, micronodules of paraffin, cement and water, but low quantities of sand, which leads to poor workability.

In reference to flexural strength, until 5% PCM can be added to Portland cement mortars without a pronounced reduction.

The required quantity of PCM in the mixture would be given by the need of latent heat required. In any case, PCM with a high heat capacity are preferred mainly, since a small quantity of PCM is able to store a substantial quantity of heat without affecting mechanical strengths.

## 5. Conclusions

To enhance the knowledge about the behaviour of Portland cement mortars mixtures containing PCM and their mix design, a number of PCM mortar mixtures with up to 15% PCM content, different w/c ratio values, and CEM I 42.5 R and CEM 52.5 R cement types were elaborated and tested.

The analysis of the gained results show that the addition of PCM made of paraffin microparticles over a silica-matrix basis to Portland cement mortars leads to a significant increase for water requirement, which causes itself the need for more cement to achieve a given resistance. For a 100 kg PCM mortar mixture additional water amounts of 40–70%, compared to non-PCM mortars mixtures, can be required. This need for water results more evident when the PCM content increase, the w/c ratio decreases, and CEM I 52.5 R is used. The required amount of cement will depend on the final amount of water used and the w/c ratio used.. Regarding the PCM content effects on the Portland cement mortars, the more PCM is used in a mixture, the lower w/c ratios are required to achieve the same compressive strength. However, for PCM contents of 15% and w/c ratios from 0.7 to 0.1, it does not significantly affect the resistance values.

Additionally, the research show that the more PCM content is used in a mixture, the less content of sand is required, which reaches zero for 15% PCM contents. This causes a reduction of the overall mixture density values of around 37% for fresh state and 45% for dry state. In general, the more grows the PCM content in a mortar, the more water absorption and open porosity increase, with values that exceed 20%. The exception are mortars with a w/c = 0.5, for which the water absorption is maintained, and open porosity may even decrease with the increase of PCM content.

Concerning the effects of PCM effect over compressive strengths, it has been stated that it is possible to reach 15.9 MPa compressive strengths for Portland cement mortars mixtures with a maximum of 15% PCM content. Additionally, if the PCM content is 5% compressive strengths beyond 40 MPa can be achieved. Although the CEM I 52.5 R cement offers greater compressive strength than CEM I 42.5 R, this cement is not always the most suitable choice, especially when greater quantities of water are required.

It has been that under certain operation conditions, it is possible to elaborate mortar mixtures which include PCM made of micronodules of paraffin over a silica-matrix basis even when the ambient temperature exceeds its change phase temperature. Therefore, the use of such PCM could increase the energy storing capacity of mortars. Nevertheless, since the flow of PCM to the mortar surface when the mixtures are in hardened state could cause stains, further research of mixtures with lower w/c ratios and PCM amounts are needed.

The results suggest also that mortars which use PCM based on paraffin microparticles in a silica-matrix basis present lower cracking tends than PCM based on microcapsules with a polymeric shell. On the other hand, the lack of the polymeric encapsulation shell may lead to a PCM, cement and water reduction within the mortar mixtures elaboration. The combination of these factors would reduce the PCM mortar cost and would improve their energy storage properties contributing to optimise the overall energy efficiency of buildings.

## Figures and Tables

**Table 1:** PCM physical properties.

Commercial Name	Core Material	Shell Material	Apparent Density (kg/m^3^)	Particles Size (mm)
PX27	Paraffin	Silica	1100	0.25
Micronal DS5001 X	Paraffin	PMMA	250–350	0.1–0.3

**Table 2:** PCM thermal properties.

Commercial Name	Phase Change Temperature (°C)	Latent Heat Capacity (kJ/kg)
PX27	24–28	117
Micronal DS5001 X	26	110

**Table 3:** Properties of cements.

Cement Type	Density (kg/m^3^)	Blaine Surface Area (cm^2^/gr)	Compressive Strength (28 Days) (MPa)
CEM I 42.5 R	3050	3750	56
CEM I 52.5 R	3050	4750	63

**Table 4:** Superplasticiser properties.

Property	Value
Type	Water-based modified polycarboxilate
Density	≈1.09 kg/cm^3^
Solids content	≈36%
pH	≈4

**Table 5:** Norms to determine materials properties.

Norm	Property
UNE-EN 1015-3:2000	Consistence of fresh mortar (by flow table)
UNE-EN 1015-10:2000	Dry bulk density of hardened mortar
UNE-EN 1015-11:2000	Flexural and compressive strength of hardened mortar
UNE-EN 1015-18:2003	Water absorption coefficient of hardened mortar
UNE-EN 196-1:2005	Strength (cement)
NCh 1564 of 2009 - Annex C	Air content (fresh concrete)

**Table 6:** Mortar mixtures compositions expressed as (kg/m^3^).

Mixture ID	w/c	Cement	PCM	Sand	SP	Water	PCM Dosage
I42.5-0.50-00-0000	0.48	454	0	1592	2.3	218	0%
I42.5-0.50-05-PX27	0.49	647	100	932	9.7	320	5%
I42.5-0.50-10-PX27	0.50	814	169	282	12.2	406	10%
I42.5-0.70-00-0000	0.68	312	0	1676	3.1	211	0%
I42.5-0.70-05-PX27	0.69	446	97	1106	6.7	308	5%
I42.5-0.70-10-PX27	0.70	587	167	510	8.8	409	10%
I42.5-0.70-15-PX27	0.70	708	212	0	10.6	495	15%
I42.5-1.00-00-0000	0.97	216	0	1764	1.1	209	0%
I42.5-1.00-05-PX27	0.99	308	98	1254	4.6	304	5%
I42.5-1.00-10-PX27	0.99	408	164	672	6.1	405	10%
I42.5-1.00-15-PX27	1.00	494	210	201	7.4	494	15%
I52.5-0.50-00-0000	0.49	444	0	1547	6.7	217	0%
I52.5-0.50-05-PX27	0.50	695	97	820	10.4	344	5%
I52.5-0.50-10-PX27	0.50	854	162	185	12.8	426	10%
I52.5-0.70-00-0000	0.68	316	0	1662	4.7	215	0%
I52.5-0.70-05-PX27	0.69	485	95	983	7.3	336	5%
I52.5-0.70-10-PX27	0.70	610	166	458	9.2	425	10%
I52.5-0.70-14-PX27	0.70	736	202	0	11.0	515	14%
I52.5-1.00-00-0000	0.97	219	0	1753	1.1	212	0%
I52.5-1.00-05-PX27	0.99	336	96	1142	5.0	332	5%
I52.5-1.00-10-PX27	0.99	428	165	630	6.4	426	10%
I52.5-1.00-15-PX27	1.00	515	209	160	7.7	515	15%

**Table 7:** Mortar mixtures compositions expressed as (kg/m^3^).

Mixture ID	w/c	Cement	PCM	Sand	SP	Water	PCM Dosage
I42.5-0.70-10-PX27	0.70	587	167	510	8.8	409	10%
I42.5-0.70-10-5001	0.69	413	181	922	6.2	289	10%

**Table 8:** Crack and fissures results of samples with different PCM.

Mixture ID	Number of Cracks and Fissures	Before Heating Thermal Tests		After Heating Thermal Tests	
		Cracks (cm)	Fissures (cm)	Cracks (cm)	Fissures (cm)
I42.5-0.7-00-0000	0	0	0	0	–
I42.5-0.7-10-5001	3	0	0	0	11
I42.5-0.7-10-PX27	0	0	0	0	–

**Table 9:** PCM content in the studied samples (expressed as kg/m^3^ of mortar).

% PCM (Weight)	CEM I 42.5 R			CEM I 52.5 R		
	w/c Ratio			w/c Ratio		
	0.5	0.7	1.0	0.5	0.7	1.0
5	100	97	98	97	95	96
10	169	167	164	162	166	165
15	-	212	210	-	209	209

**Table 10:** Influence of PCM content on compressive strength.

Cement Type	w/c	PCM Content	Compressive Strength (MPa)
CEM I 42.5 R	0.5	0%	45.8
CEM I 52.5 R			50.0
CEM I 42.5 R		5%	36.2
CEM I 52.5 R			41.4
CEM I 42.5 R	1	0%	11.3
CEM I 52.5 R			15.9
CEM I 42.5 R		15%	12.4
CEM I 52.5 R			13.0

## Data Availability

Data available on request to the corresponding author.

## Nomenclature

$A_{c}$	estimated air content in fresh mortar, %
$O_{p}$	open porosity, %
$P_{d}$	dry weight (in a stove at 40 °C), g
$P_{s}$	saturated weight, g
$P_{ss}$	immersed weight, g
$V_{a}$	apparent volume of fresh mortar, m^3^
$V_{c}$	real cement volume, m^3^
$V_{d}$	real admixture volume, m^3^
$V_{PCM}$	real PCM volume, m^3^
$V_{r}$	real volume of fresh mortar, m^3^
$V_{w}$	real water volume, m^3^
$W_{a}$	water absorption coefficient, %

## Abbreviations

The following abbreviations are used in this manuscript:

PCM	Phase change materials
MPCM	Microencapsulated phase change materials
GPC	Geopolymer concretes
PCC	Portland cement concretes
FBA	Forest biomass ashes
CCPCMs	Cement-based composite phase change materials
PEG	Polyethylene glycol
SP	Superplasticiser

## References

[1] Wang X., Zhang Y., Xiao W., Zeng R., Zhang Q., Di H. (2009). Review on thermal performance of phase change energy storage building envelope. Chin. Sci. Bull..

[2] Akeiber H., Nejat P., Majid M.Z.A., Wahid M.A., Jomehzadeh F., Famileh I.Z., Calautit J.K., Hughes B.R., Zaki S.A. (2016). A review on phase change material (PCM) for sustainable passive cooling in building envelopes. Renew. Sustain. Energy Rev..

[3] Memon S.A. (2014). Phase change materials integrated in building walls: A state of the art review. Renew. Sustain. Energy Rev..

[4] Tyagi V.V., Buddhi D. (2007). PCM thermal storage in buildings: A state of art. Renew. Sustain. Energy Rev..

[5] Sharma A., Tyagi V., Chen C., Buddhi D. (2009). Review on thermal energy storage with phase change materials and applications. Renew. Sustain. Energy Rev..

[6] Oliver A., Neila González F.J., García-Santos A. (2012). Clasificación y selección de materiales de cambio de fase según sus características para su aplicación en sistemas de almacenamiento de energía térmica. Mater. Constr..

[7] Marani A., Nehdi M.L. (2019). Integrating phase change materials in construction materials: Critical review. Constr. Build. Mater..

[8] Berardi U., Gallardo A.A. (2019). Properties of concretes enhanced with phase change materials for building applications. Energy Build..

[9] Rathore P.K.S., Shukla S.K. (2020). An experimental evaluation of thermal behavior of the building envelope using macroencapsulated PCM for energy savings. Renew. Energy.

[10] Sun X., Jovanovic J., Zhang Y., Fan S., Chu Y., Mo Y., Liao S. (2019). Use of encapsulated phase change materials in lightweight building walls for annual thermal regulation. Energy.

[11] Zhang Y., Zhou G., Lin K., Zhang Q., Di H. (2007). Application of latent heat thermal energy storage in buildings: State-of-the-art and outlook. Build. Environ..

[12] Ling T.C., Poon C.S. (2013). Use of phase change materials for thermal energy storage in concrete: An overview. Constr. Build. Mater..

[13] Zhang D., Zhou J., Wu K., Li Z. (2005). Granular phase changing composites for thermal energy storage. Sol. Energy.

[14] Lee T., Hawes D., Banu D., Feldman D. (2000). Control aspects of latent heat storage and recovery in concrete. Sol. Energy Mater. Sol. Cells.

[15] Pongsopha P., Sukontasukkul P., Phoo-ngernkham T., Imjai T., Jamsawang P., Chindaprasirt P. (2019). Use of burnt clay aggregate as phase change material carrier to improve thermal properties of concrete panel. Case Stud. Constr. Mater..

[16] Bentz D., Peltz M., Durán-Herrera A., Valdez P., Juárez C. (2011). Thermal properties of high-volume fly ash mortars and concretes. J. Build. Phys..

[17] Du Y., Liu P., Quan X., Ma C. (2020). Characterization and cooling effect of a novel cement-based composite phase change material. Sol. Energy.

[18] Xie N., Niu J., Zhong Y., Gao X., Zhang Z., Fang Y. (2020). Development of polyurethane acrylate coated salt hydrate/diatomite form-stable phase change material with enhanced thermal stability for building energy storage. Constr. Build. Mater..

[19] Essid N., Eddhahak-Ouni A., Neji J. (2020). Experimental and Numerical Thermal Properties Investigation of Cement-Based Materials Modified with PCM for Building Construction Use. J. Archit. Eng..

[20] Rathore P.K.S., Shukla S.K., Gupta N.K. (2020). Potential of microencapsulated PCM for energy savings in buildings: A critical review. Sustain. Cities Soc..

[21] Zetola Vargas V., García Santos A., Neila González F.J. (2013). Mortero de cemento Portland con parafinas microencapsuladas. Rev. Constr..

[22] Jayalath A., San Nicolas R., Sofi M., Shanks R., Ngo T., Aye L., Mendis P. (2016). Properties of cementitious mortar and concrete containing micro-encapsulated phase change materials. Constr. Build. Mater..

[23] Haurie L., Serrano S., Bosch M., Fernández A.I.F., Cabeza L.F. Addition of microencapsulated PCM into single layer mortar: Physical and thermal properties and fire resistance. Proceedings of the Eurotherm Seminar #99: Advances in Thermal Energy Storage [s. n.].

[24] Norvell C., Sailor D.J., Dusicka P. (2013). The effect of microencapsulated phase-change material on the compressive strength of structural concrete. J. Green Build..

[25] Cunha S., Lima M., Aguiar J.B. (2016). Influence of adding phase change materials on the physical and mechanical properties of cement mortars. Constr. Build. Mater..

[26] Xu B., Li Z. (2013). Paraffin/diatomite composite phase change material incorporated cement-based composite for thermal energy storage. Appl. Energy.

[27] Li X., Sanjayan J.G., Wilson J.L. (2014). Fabrication and stability of form-stable diatomite/paraffin phase change material composites. Energy Build..

[28] Zhang Z., Shi G., Wang S., Fang X., Liu X. (2013). Thermal energy storage cement mortar containing n-octadecane/expanded graphite composite phase change material. Renew. Energy.

[29] Li H., Chen H., Li X., Sanjayan J.G. (2014). Development of thermal energy storage composites and prevention of PCM leakage. Appl. Energy.

[30] Pilehvar S., Cao V.D., Szczotok A.M., Valentini L., Salvioni D., Magistri M., Pamies R., Kjøniksen A.L. (2017). Mechanical properties and microscale changes of geopolymer concrete and Portland cement concrete containing micro-encapsulated phase change materials. Cem. Concr. Res..

[31] Farinha C.B., de Brito J., Veiga R. (2019). Influence of forest biomass bottom ashes on the fresh, water and mechanical behaviour of cement-based mortars. Resour. Conserv. Recycl..

[32] Kuznik F., David D., Johannes K., Roux J.J. (2011). A review on phase change materials integrated in building walls. Renew. Sustain. Energy Rev..

[33] Da Cunha S.R.L., de Aguiar J.L.B. (2020). Phase change materials and energy efficiency of buildings: A review of knowledge. J. Energy Storage.

[34] Cunha S., Leite P., Aguiar J. (2020). Characterization of innovative mortars with direct incorporation of phase change materials. J. Energy Storage.

[35] Rubitherm. https://www.rubitherm.eu/en/index.php/productcategory/organische-pcm-rt.

[36] BASF (2008). Micronal® PCM Intelligent Temperature Management for Buildings.

[37] AENOR (2011). UNE-EN 197-1:2011-Cement—Part 1: Composition, Specifications and Conformity Criteria for Common Cements.

[38] SIKA (2005). Viscocrete 5720 Technical Data Sheet.

[39] AENOR (2000). UNE-EN 1015-3:2000, Methods of Test for Mortar for Masonry—Part 3: Determination of Consistence of Fresh Mortar (by Flow Table).

[40] AENOR (2005). UNE-EN 196-1:2005—Methods of Testing Cement—Part 1: Determination of Strength.

[41] AENOR (2000). UNE-EN 1015-10:2000, Methods of Test for Mortar for Masonry. Part 10: Determination of Dry Bulk Density of Hardened Mortar.

[42] AENOR (2003). UNE EN 1015-18:2003, Methods of Test for Mortar for Masonry—Part 18: Determination of Water Absorption Coefficient due to Capillary Action of Hardened Mortar.

[43] Mayor Lobo P.L., Hernández Olivares F. (2007). Determinación por procedimientos físico-mecánicos de la dosificación de agua en morteros monocapa. Su incidencia en la aparición de patologías en la obra terminada. Inf. Constr..

[44] Instituto Nacional de Normalización de Chile—INN Chile (2009). NCh1564—Hormigón—Determinación de la Densidad Aparente del Hormigón Fresco.

[45] AENOR (2000). UNE-EN 1015-11:2000, Methods of Test for Mortar for Masonry—Part 11: Determination of Flexural and Compressive Strength of Hardened Mortar.

[46] American Concrete Institut (ACI) Committee 305 (1999). Hot Weather Concreting.

